# From stridor to symptom relief; management of pediatric Kommerell's diverticulum and vascular rings in Palestine [2016–2024]: a single center cohort study

**DOI:** 10.3389/fped.2025.1713368

**Published:** 2025-12-05

**Authors:** Mohammad Amouri, Salahaldeen Deeb, Nour Sourkhi, Sara Qasim, Boshra Abu Hadeed, Khalil Omar Atatra, Ahmad Nasereddin

**Affiliations:** 1 Faculty of Medicine, Al-Quds University, Jerusalem, Palestine; 2 ENT Head and Neck Surgery Department, Rafedia Surgical Hospital, Nablus, Palestine

**Keywords:** Kommerell’s diverticulum, vascular rings, stridor, dysphagia lusoria, aortopexy, aberrant subclavian artery, right aortic arch

## Abstract

**Background:**

Kommerell's diverticulum (KD) with vascular rings is an uncommon cause of pediatric airway and feeding symptoms, and data from the Arab region are scarce. We report a Palestinian cohort of all diagnosed and managed cases, describing presentation, diagnostics, and management.

**Methods:**

We retrospectively studied all children at Al-Makassed Hospital (Jerusalem) from 2016 to 2024 who were diagnosed with vascular rings + KD confirmed by imaging or intra-operative findings. We abstracted demographics, symptoms, test performance, anatomy, treatments, and peri-operative course and follow-up. Primary outcomes were diagnostic yield and improvement at last visit.

**Results:**

Fourteen patients were included; median age at diagnosis was 8 months (IQR 2–15), symptom onset 15 days (0–83). Thirteen (92.9%) were symptomatic; stridor (64.3%), recurrent wheeze (35.7%), and dysphagia/choking (28.6%) predominated; 42.9% had prior misdiagnoses. Barium swallow was positive in 7/8 (87.5%), echocardiography in 5/9 (55.6%), bronchoscopy in 10/12 (83%), and CT angiography (CTA) confirmed anatomy in 13/13 (100%). Anatomy included right aortic arch (RAA) with aberrant left subclavian artery in 8/14 (57.1%), double aortic arch in 4/14 (28.6%), and innominate artery compression in 2/14 (14.3%); 57.1% had minor intracardiac anomalies. Thirteen children (92.9%) underwent surgery (ligamentum division, diverticulectomy ± subclavian transfer, aortopexy, or double-arch division); one mild innominate compression case was observed. All were discharged alive; one late death occurred. At last follow-up, 7/13 survivors were asymptomatic and 6/13 had residual but improved symptoms. Median hospital stay was 18 days (IQR 7–25).

**Conclusion:**

KD-associated rings present early yet are frequently delayed in diagnosis. CTA is definitive, and tailored surgery yields meaningful, often complete, symptom relief with acceptable morbidity.

## Introduction

Kommerell's diverticulum (KD) is a rare congenital vascular anomaly, first described by the radiologist Burckhard Kommerell in 1936. His initial observation was in a patient with dysphagia due to esophageal compression related to an aneurysmal origin of an aberrant right subclavian artery (ARSA) arising from a left-sided aortic arch. Currently, the definition includes a localized aneurysmal dilation at either the right- or left-sided aortic arch at the origin of an aberrant subclavian artery (1).

KD patients often remain asymptomatic; they are diagnosed incidentally in adults, with a mean age of about 56 years (range 18–91) (2). Even though many adults remain asymptomatic, symptomatic patients, typically 30%–40% of cases, can experience a range of tracheoesophageal symptoms. These may include dysphagia, stridor, recurrent respiratory infections, and chest pain. Adults may experience symptoms later in life due to arteriosclerosis and decreased esophageal compliance, which increases compression (3). On the other hand, pediatric patients are often diagnosed earlier because of compression airway symptoms (4). Untreated symptomatic KD can result in persistent symptoms, whereas asymptomatic cases may remain undetected until adulthood (1). The severity of clinical manifestations can depend on factors like the size of the diverticulum and its proximity to the trachea and esophagus (1). KD diagnosis is confirmed by computed tomography (CT) and magnetic resonance imaging (MRI), which are the most reliable diagnostic modalities for identifying associated arch anomalies. Surgical interventions are indicated only for patients presenting with severe esophageal or tracheal compression, while their use is controversial for asymptomatic KD (5).

Despite the increasing recognition of KD in recent medical literature, with several international case series and reviews documenting clinical presentations, diagnostic approaches, and surgical outcomes, existing reports remain scarce and haven't filled knowledge gaps, particularly in pediatric patients. To date, no published data from Palestine describe the clinical presentation, management strategies, or long-term outcomes of KD with vascular rings in children.

Our retrospective, single-center study represents the first publication from Palestine on pediatric patients with KD. We reviewed 14 pediatric cases with (KD), thus addressing a population that has been underrepresented in existing literature. Our study provides an assessment of the clinical presentation, diagnostic strategies, surgical interventions, and follow up outcomes in pediatric patients. This establishes a foundation for further research on KD in Palestine and across the Arab region.

## Patients and methods

### Study design and setting

This retrospective observational cohort study was conducted at Al-Makassed Hospital, east Jerusalem, covering the period 1 January 2016 to 31 December 2024. Reporting follows the STROBE statement for observational studies. During the study period, suspected vascular rings were evaluated by a multidisciplinary team in pediatric cardiology, cardiothoracic surgery, radiology, and pulmonology.

### Ethical approval

The protocol was approved by the Al-Makassed Hospital Research Ethics Committee and the Institutional Review Board (IRB) of Al-Quds University [411/REC/2024]. Because this was a retrospective chart review using de-identified data, both committees waived the requirement for individual informed consent.

### Patient population and eligibility criteria

We included all consecutive children evaluated at our institution within the study window who had a vascular ring with or without KD confirmed by cross-sectional imaging and/or intra-operative findings. We excluded patients with incomplete records that precluded confirmation of ring anatomy, encounters outside the time window, and duplicate records. Antenatal diagnoses were retained in the overall cohort but excluded from analyses that require postnatal intervals (e.g., symptom-to-diagnosis).

### Definitions and anatomical classification

A KD was defined as a diverticular origin of an aberrant subclavian artery from the aortic arch or descending aorta. Vascular ring subtypes were classified *a priori* from imaging and operative reports (e.g., right aortic arch (RAA) with aberrant left subclavian artery (ALSA) ± KD; double aortic arch; innominate artery compression; other aberrant subclavian variants). “Diagnostic positivity” for each test followed modality-specific criteria: extrinsic indentation on barium swallow; pulsatile airway compression on bronchoscopy; arch or branch-vessel anomaly on echocardiography; and definitive ring anatomy on contrast-enhanced CT angiography (CTA).

### Data sources and variables

Data were abstracted from the electronic medical record, radiology PACS, bronchoscopy reports, operative and anesthesia records, and outpatient notes. Baseline variables included sex, antenatal detection, age at symptom onset, diagnosis, and intervention, presenting respiratory and gastrointestinal symptoms, prior misdiagnoses, and comorbidities. Diagnostic work-up captured the use and results of chest radiography, barium swallow, transthoracic echocardiography, bronchoscopy, and contrast-enhanced CT; MRI or catheterization were recorded if performed, but neither was used as a primary diagnostic modality in this cohort. Anatomic variables detailed ring subtype and associated intracardiac lesions (e.g., ASD, VSD, PFO, bicuspid aortic valve). Treatment variables included operative approach (thoracotomy vs. sternotomy), procedures (e.g., ligamentum arteriosum division, KD resection/diverticulectomy, subclavian reimplantation/transfer, aortopexy, double-arch division), and use of cardiopulmonary bypass where applicable. Peri-operative course (ICU and total length of stay), post-operative complications, reinterventions (planned or completed), and vital status were recorded. Symptom status at last follow-up and growth/nutrition (when available) served as clinical outcomes.

### Outcomes and endpoints

Primary outcomes were: (1) diagnostic yield of each evaluation modality and (2) clinical improvement after management, defined as resolution or reduction of ring-related respiratory/feeding symptoms at last follow-up. Secondary outcomes included peri-operative complications, length of hospital stay, mortality (in-hospital and late), and the need for reintervention.

### Data abstraction and quality assurance

A standardized data-extraction template was piloted and then applied to all eligible charts. Discrepancies across sources (e.g., imaging vs. operative notes) were resolved by consensus after reviewing the original reports. All dates were recorded in day-month-year format and cross-checked to ensure temporal consistency among symptom onset, diagnosis, and intervention.

### Handling of missing data

Unavailable variables were coded as missing and reported with variable-specific denominators. Analyses of modality-specific diagnostic yield and temporal intervals used complete-case data. By design, antenatally diagnosed cases were excluded from postnatal interval calculations. KD diameter was abstracted where available but not used for stratification due to non-standardized measurement across the study period.

### Statistical analysis

Continuous variables are summarized as median [interquartile range (IQR)] and categorical variables as counts (percentages). Given the small, heterogeneous sample, between-group comparisons were considered exploratory (Fisher's exact test for categorical data; Mann–Whitney *U* test for continuous data; two-sided *α* = 0.05). Analyses were performed in R (R Foundation for Statistical Computing) or equivalent validated software. Figures (e.g., diagnostic positivity proportions, patient-flow diagram) were generated from the cleaned analytic dataset.

### Bias and confounding

Selection bias was minimized by including all consecutive eligible patients. Information bias was mitigated by multi-source verification (radiology, bronchoscopy, and operative documentation). Potential confounding by anatomical subtype and concomitant congenital heart disease was explored descriptively; formal multivariable modeling was not undertaken due to sample size constraints.

### Data security and confidentiality

Data were de-identified prior to analysis. The master linkage file was stored on a password-protected institutional server accessible only to the study team. All procedures adhered to institutional policies and the approvals granted by Al-Makassed Hospital and the Al-Quds University IRB.

### Transparency and reproducibility

A variable dictionary and analysis codebook are available from the corresponding author upon reasonable request and with permission of the approving ethics committees.

## Results

### Cohort characteristics

Fourteen patients met inclusion criteria, all with a vascular ring associated with a KD. Median age at diagnosis was 8 months (IQR 2–15 months), with symptoms typically manifesting in early infancy (median onset 15 days [0–83 days]). The cohort was evenly split by sex (7 males, 50%). All but one patient (13/14, 92.9%) were symptomatic at presentation, most commonly with respiratory distress (stridor in 64.3%, recurrent wheezing in 35.7%) or feeding difficulties (dysphagia/choking in 28.6%), one patient (7.1%) was asymptomatic, with the vascular ring incidentally identified during workup for an unrelated cardiac condition, as shown in Table 1. Nearly half of the cohort (6/14, 42.9%) had been previously misdiagnosed with more common conditions (e.g., recurrent pneumonia or reactive airway disease) before the correct diagnosis was established. Notably, the time interval from symptom onset to diagnosis was recorded in 12 patients (median 3.53 months [0.62–12.65 months]) reflecting a variable diagnostic delay and was not systematically available for the remainder, Two patients were diagnosed antenatally and were therefore excluded from the postnatal symptom-to-diagnosis interval analysis.

**Table 1:** Summary of clinical preoperative characteristics and symptoms.

Patient no.	Sex	Respiratory symptoms	Gastrointestinal symptoms	Other symptoms	Associated cardiac anomalies
1	M	Stridor	…	Failure to thrive & cyanosis	…
2	M	…	…	…	ASD
3	M	Stridor	Choking	Failure to thrive	ASD & VSD
4	M	Stridor, recurrent wheezing & cough	…	…	VSD
5	F	Cough	…	Failure to thrive & cyanosis	ASD
6	M	Stridor	Dysphagia	…	PFO
7	F	Recurrent wheezing & cough	…	…	…
8	F	Recurrent wheezing	Choking	Cyanosis	VSD, ASD & PFO
9	M	Stridor & cough	Dysphagia & choking	Failure to thrive	Bicuspid aortic valve, ASD & VSD
10	M	Stridor & cough	…	…	VSD
11	F	Stridor & cough	Dysphagia & choking	…	…
12	F	Stridor & cough	…	…	…
13	F	Stridor, recurrent wheezing & cough	Dysphagia & choking	Cyanosis	…
14	F	Stridor, cough	Choking	Failure to thrive	PFO

M, male; F, female; ASD, atrial septal defect; VSD, ventricular septal defect; PFO, patent foramen ovale.

### Diagnostic workup and findings

**Figure 1:**
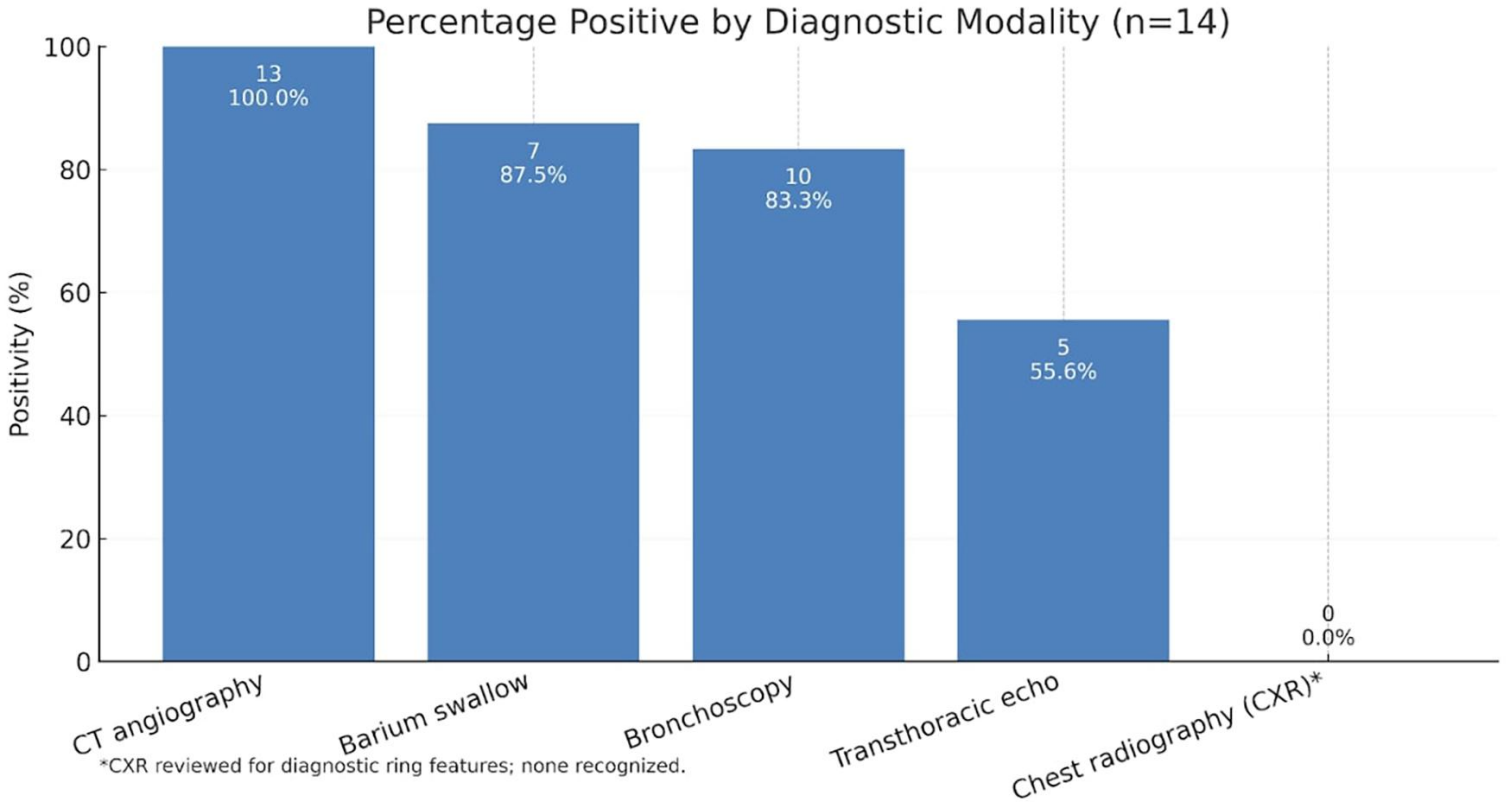
Overview of percentage positive by diagnostic modalities used in our cohort of KD-associated vascular rings.

All patients underwent chest imaging and cross-sectional evaluations as part of their workup. Chest radiography (CXR) suggested no diagnostic ring features were recognized in all patients. Barium swallow studies were performed in 8 patients (typically those with feeding symptoms) and demonstrated extrinsic esophageal compression in 7/8 (87.5%). Transthoracic echocardiography, obtained in 9 patients (64.3%), identified an arch anomaly in 5/9 (55.6%) mainly among those with a RAA and also screened for intracardiac defects [3 patients had small intracardiac anomalies such as atrial septal defect (ASD) or ventricular septal defect (VSD)]. Diagnostic bronchoscopy was utilized in 12 patients (85.7%), nearly always confirming pulsatile tracheal compression; 10/12 (83%) had visible airway indentation by the ring, often with concurrent tracheomalacia (noted in 4 cases, 28.6%) or bronchomalacia (1 case). Contrast-enhanced CTA was the definitive diagnostic modality in this cohort. Thirteen patients (92.9%) underwent CT, which confirmed the vascular ring anatomy in 100% of those imaged (The remaining patient's diagnosis was confirmed intra-operatively during open heart surgery for another indication). MRI or cardiac catheterization were not used for diagnosis in any case. Figure 1 illustrates the positivity rates of each diagnostic modality.

In 13 patients (92.9%), the vascular ring was first suspected by non-invasive imaging (barium swallow in 7, echocardiogram in 5, and/or bronchoscopy in 7), whereas in one asymptomatic case (7.1%) the ring was an unexpected operative finding.

#### Anatomy of vascular rings

The majority of patients (8/14, 57.1%) had a RAA with an ALSA all of whom by definition concealed a KD at the origin of the aberrant subclavian. Four patients (28.6%) had a double aortic arch, and the remaining two (14.3%) had an innominate artery compression.

Additional congenital cardiac anomalies were present in eight patients (57.1%): ASD (*n* = 3), VSD (*n* = 5), patent foramen ovale (PFO) (*n* = 3), and/or bicuspid aortic valve (*n* = 1). None altered management. Table 1 details the full distribution of these anomalies.

### Treatment modalities

**Figure 2:**
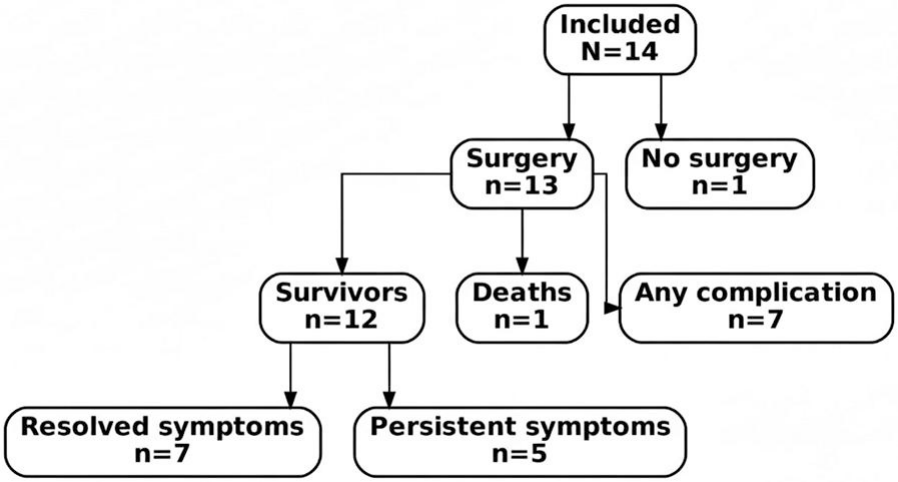
Patient flow diagram summarizing inclusion, management pathways (surgery vs. no surgery), and outcomes.

Thirteen patients (92.9%) underwent surgical ring division and repair, whereas one symptomatic child (7.1%) was managed conservatively during the study period. The single non-operative case was an infant with an innominate artery compression ring who had mild symptoms; this patient remained under observation without surgery. Figure 2 depicts the patient flow through the study, including the distribution of symptomatic vs. asymptomatic cases and their management and outcomes. All patients who underwent surgery had open surgical correction via thoracotomy; no cases were managed with primary endovascular stenting or other purely endoluminal techniques. In one asymptomatic infant, the ring was detected and divided during surgery for another cardiac anomaly. No intra-operative mortalities occurred. Median age at repair was 13 months [3–26 months], reflecting a tendency to intervene early in life for severe forms (e.g., median 6 months in double aortic arch) and somewhat later in aberrant subclavian rings (median ∼15 months for right-arch ALSCA patients). The decision to operate in the one asymptomatic case was made in light of the ease of ring division during the concurrent procedure and the presence of a sizable diverticulum.

#### Surgical management

Thirteen of fourteen children (92.9%) underwent operative repair. The procedures used in our cohort comprised four standard approaches as shown in Table 2. (1) Ligamentum arteriosum division of the ductal remnant/ligamentum to open the vascular ring; this is the least extensive operation and is typically reserved for rings without a prominent diverticular pouch. (2) KD resection excision of the posterior diverticular pouch that tethers/compresses the esophagus and airway, commonly performed with or without reimplantation of the aberrant subclavian artery (e.g., transfer to the carotid/subclavian) to eliminate a residual “posterior mass effect” and straighten the course of the vessel. (3) Aortopexy an adjunctive airway-decompression maneuver in which the ascending aorta (or arch) is suspended to the posterior sternum to relieve anterior tracheal compression and address associated tracheomalacia; in KD-related rings this is used when dynamic collapse or anterior compression persists despite ring division/diverticulectomy. (4) Double-arch division for double aortic arch anatomy, division of the non-dominant arch together with the ligamentum to break the ring (with intraoperative confirmation of dominant arch perfusion). One child was managed non-operatively at the time of last follow-up.

**Table 2:** Shows the surgical description and outcome for our cohort patients.

Patient no.	Sex	Age at surgery	Type	Description of surgery	Persistent symptoms	Complications	Hospitalisation (days)
1	M	2 y 10 mo	3	Aberrant left subclavian artery re-implantation, kommeral diverticulum excision and PDA devision	None	Sepsis, pneumothorax, subcutaneous emphysema & chylothorax.	20
2	M	26 days	3	Artopexy	None	None	4
3	M	2 mo	1	Left aortic arch division and secured	None	None	17
4	M	2 y 4 mo	3	PDA ligation and division, aortopexy	None	None	6
5	F	2 y	3	Ligamentum arteriosum division and fine dissection around trachea with release of ring	None	None	7
6	M	4 mo	1	PDA devision	Stridor	None	18
7	F	1 y 5 mo	3	Dissection aortic arch vessels, PDA Devision, Kommeral diverticulum devision (anterior aortopexy)	None	Atelactasis	21
8	F	3 mo	3	PDA division and reimplantation of left subclavian artery of double aortic arch	Stridor	Sepsis & pneumothorax	38
9	M	6 y 3 mo	4	PDA Division, ligamentum arteriosum	Choking	None	34
10	M	8 mo	1	PDA devision	Choking	Cardiac arrest & pneumothorax	25
11	F	-	4	No surgery	None	None	-
12	F	2 y 2 mo	3	PDA division and Aortopexy	None	Pneumothorax	6
13	F	1 y 1 mo	3	PDA devision	Cough	Recurrent respiratory infections	39
14	F	10 mo	1	PDA devision	Stridor	Pericardiac effusion, subcutaneous hematoma	23

M, male; F, female; mo, month; Y, year; N, none; Y, yes; 1, double aortic arch; 2, pulmonary artery sling; 3, right aortic arch with left aberrant subclavian artery; 4, innominate artery compression; 5, Aberrant right subclavian. “None” in a given column indicates the absence of that specific item (e.g., “None” under persistent symptoms = no persistent symptoms; “None” under complications = no complications).

Operative approach varied by anatomy and planned reconstruction: posterolateral thoracotomy (left for LAA/ARSA, right for RAA/ALSA) was used for isolated ligamentum division or diverticulectomy, whereas median sternotomy was selected when aortopexy or subclavian reimplantation was anticipated. Cardiopulmonary bypass was not routinely required for simple ligament division or diverticulectomy, but was available when arch reconstruction or complex reimplantation was planned. Overall, the operative repertoire reflects a step-up strategy tailored to mechanism of compression simple ring division in select cases vs. KD resection ± subclavian transfer and/or aortopexy when a posterior pouch and/or dynamic airway collapse were contributory.

Additionally, two other patients (14.3%) were documented to have plans for reintervention at the time of last follow-up (one for persistent tracheomalacia requiring aortopexy, and one for a residual diverticular remnant planned for excision), but these had not yet been performed during the study period. Notably, KD diameter was not systematically measured in this cohort's records, so no analysis of diverticulum size thresholds was possible.

### Outcomes

All 14 patients were alive at hospital discharge. There was one late death (7.1%) in the cohort. Excluding that mortality, 13/14 (92.9%) survived to the latest follow-up. Among survivors, 7/13 (53.8%) were free of any residual symptoms attributable to the vascular ring at last evaluation. The other 6/13 (46.2%) continued to have some degree of residual airway or esophageal symptoms (primarily mild stridor or choking). In the single patient managed non-operatively, the compressive symptoms persisted but remained stable under observation.

Post-operative complications in the surgical cohort (*n* = 13) are reported in Table 2 as events, recognizing that patients could experience and/or more than one complication. Pneumothorax was most frequent (4), followed by sepsis (2). Single-event complications (each *n* = 1) comprised atelectasis, subcutaneous emphysema, chylothorax, recurrent respiratory infections, pericardial effusion, and subcutaneous hematoma. The overall median length of hospital stay for patients who underwent surgery was 18 days [7–25 days]; one patient, who had the longest hospital stay in the cohort (25 days, including time in the intensive care unit), was discharged in stable condition with persistent symptoms; specifically, choking. He had a known VSD and had experienced postoperative complications including pneumothorax and a cardiac arrest. A reintervention was planned; however, the patient died at home before the elective procedure could be performed. This was the only death in the cohort.

Despite the relatively high rate of initial post-operative morbidity, the long-term outcomes were favorable in most cases. Half of the operated patients were asymptomatic and thriving with normal growth. As noted above, about 46% had minor residual symptoms; importantly, these were markedly improved compared to pre-operative status in all but one case. The single non-operated patient continued to be monitored, with symptoms unchanged (and a plan for elective surgery if any progression occurs). Notably, all patients who underwent surgery experienced at least partial relief of their compressive symptoms, whereas the patient who did not undergo surgery did not improve. At last follow-up, 90% of the survivors were free of any debilitating ring-related symptoms, and growth percentiles had normalized in all but one child (who had feeding difficulty due to neurologic comorbidities).

No significant differences in outcomes were discernible between different anatomical subtypes in this small cohort. Patients with double aortic arch tended to present earlier, but with appropriate surgical division they achieved symptom resolution rates comparable to those with aberrant subclavian rings. The single-center nature and limited sample size precluded formal statistical comparison, but the data hint that innominate artery compression syndrome may be more amenable to conservative management in milder cases.

## Discussion

In our single center cohort study we include the diagnostic and surgical modalities as well as the clinical outcomes of children with vascular rings, in association with KD. Based on the results above, we have found significant multi-aspects findings: The delay in diagnosis accompanying these anomalies, the golden role of using CTA as a diagnostic test, surgery as a preferable treatment and long term outcomes.

Most Adult patients with KD are asymptomatic, and often diagnosed incidentally as reported in the literature (3). In contrast, nearly all patients in our cohort were symptomatic (92.9%) this difference stems from the fact that our patients are pediatric. The predominant manifestations were respiratory, including stridor (64.3%) and recurrent wheezing (35.7%), reflecting tracheal compression from the diverticulum and aberrant subclavian artery. Feeding difficulties, such as dysphagia and choking, were observed in 28.6% of patients and were consistent with esophageal compression. This rate is notably higher than that reported in other vascular ring series, where dysphagia occurs in only 3%–12% of cases, suggesting that KD exerts a greater mass effect (6). Dysphagia lusoria has been described as a characteristic symptom in cases with marked esophageal compression, as highlighted by Tanaka et al. (4). Differences in presentation across age groups have also been reported: in adults, van Rosendael et al. described dysphagia as the dominant presentation, whereas in pediatric cohorts, respiratory distress tends to predominate (1, 4). One patient in our cohort was asymptomatic, with the vascular ring discovered incidentally during evaluation for an unrelated cardiac condition.

In our cohort, the majority of patients (57.1%) presented with RAA and ALSA, 28.6% had double aortic arch and 14.3% with innominate artery compression. These distributions are consistent with previously reported series, where the RAA with ALSA is recognized as the most common anatomic variant associated with KD (4, 6). The presence of other congenital heart anomalies in over half of our patients including ASD, VSD, PFO, and bicuspid aortic valve further necessitates the need for comprehensive evaluation in this population. Although none of these anomalies altered surgical management in our series, other studies have emphasized that concomitant defects may complicate perioperative care or influence the timing of intervention (3, 7). This overlap between vascular rings and intracardiac anomalies underscores the importance of multimodal imaging and multidisciplinary assessment in infants with suspected KD.

The golden standard in diagnosis of vascular rings associated with KD is CT, precisely CTA with three dimensional reconstruction. It provides detailed knowledge of the nature of the anomaly, including the diverticulum on its own as well as the associated aberrant subclavians. Furthermore, the ability to differentiate the type of the aneurysm (KD vs. true/pseudoanurysm) (6). In this cohort study, contrast-enhanced CTA was the definitive diagnostic modality; 92.9% of our sample patients underwent CT, which confirmed the vascular ring anatomy in 100% of those imaged. Traditional imaging methods used previously to diagnose vascular ring anomaly; such as barium swallow studies, has been replaced to a larger range by CTA; this is due to the latter's ability to multi-visualize both vascular and airway anatomy at the same time (8). In addition, more information like the size of the aneurysm, the degree of compression on both the esophagus and trachea, are of particular importance in clinical decision making and determination of surgery (1). Putting all these facts together, CT imaging leads the process of diagnosis and treatment to a further point.

Surgical intervention was performed in 92.9% of our patients, while one case was managed conservatively due to mild symptoms. a pattern that contrasts with adult right-sided arch series in which most KD are surveilled unless symptomatic or enlarging, e.g., van Rosendael et al. reported surgery in just 1/7 adults and suggested intervention for symptoms or when KD diameter exceeds ∼30 mm (1). The operative approach varied by anatomy and the planned reconstruction was individualized according to the presence of a KD. Procedures performed were complex and included division of the ligamentum arteriosum, diverticulectomy with or without aberrant subclavian artery reimplantation, aortopexy for persistent airway compression, and double-arch division when indicated. Aligns with historical pediatric vascular-ring practice favoring muscle-sparing thoracotomy and, for KD with aberrant subclavian artery, diverticulectomy plus carotid-subclavian transfer, and with contemporary reviews emphasizing individualized reconstruction based on arch anatomy and compressive symptoms (4, 8).

The choice between ligation and division of an aberrant subclavian artery (±KD plication/exclusion) vs. carotid reimplantation must balance simplicity against long-term perfusion and neurologic safety. Ligation and division through a 4th intercostal muscle-sparing thoracotomy offers a technically straightforward option in patients with a diminutive KD, absent airway or esophageal compression, and adequate collateral arm perfusion. This strategy has been historically justified by analogies to subclavian flap aortoplasty, where ipsilateral upper-limb perfusion is preserved through native collaterals (8, 9). However, such analogies have limitations, especially in cases with ipsilateral vertebral artery dominance or signs of posterior circulation vulnerability. In contrast, carotid reimplantation or subclavian-to-carotid transposition remains the preferred approach in patients with a large or aneurysmal KD, significant tracheoesophageal compression, or concern for antegrade vertebral flow compromise (2, 10). Although reimplantation involves more extensive mediastinal dissection and carries risks such as chylothorax or neurologic complications, modern pediatric series report acceptable morbidity with careful technique (4). Moreover, histologic degeneration has been identified in many resected diverticula, supporting the practice of definitive resection and revascularization in selected cases (2). We advocate for preoperative assessment of vertebral dominance, perfusion asymmetry, and KD morphology to guide individualized surgical planning. In summary, ligation and division may be acceptable in anatomically favorable patients with minimal KD and robust collaterals, while reimplantation is preferred when vertebral dominance, significant KD, or long-term antegrade flow concerns are present.

Most surgeries were performed via thoracotomy, while median sternotomy was reserved for patients requiring more extensive reconstruction or aortopexy. whereas a recent multicenter adult cohort (*n* = 285) used hybrid strategies in 59%, open in 36%, and endovascular-only in 5%; that study reported indications driven by symptoms (59%) and size (38%), a median postoperative length of stay of 11 days, >80% symptom relief, and proposed considering repair at ∼40 mm (6). Our longer median hospital stay after these procedures was 18 days, reflecting the higher rate of complex airway procedures (aortopexy) and double-arch division, which are uncommon in adult series but typical in ring-related airway compression.

Postoperative complications were relatively frequent, pneumothorax being the most common, followed by sepsis, and other complications, including atelectasis, chylothorax, subcutaneous emphysema, pericardial effusion, and subcutaneous hematoma. Mirroring the pleural, lymphatic, and nerve-related morbidities described after surgery for KD and vascular rings (4, 8, 11). We observed no intraoperative deaths and a single postoperative mortality (7.1%) due to cardiac arrest; while higher than the near-zero operative mortality typically reported in pediatric-predominant vascular-ring series (8). The majority of patients who underwent surgery experienced resolution or significant improvement of compressive symptoms over the long term. These outcomes are consistent with prior studies that emphasize the importance of timely, anatomy-guided surgical correction of KD-associated vascular rings to relieve airway and feeding difficulties, and similarly show a modest risk of early postoperative morbidity (4, 8).

### Limitations

This study has two main limitations: first, the single centered small sample size and second, the lack of systematic measurement of the KD size and long term follow ups. We encourage healthcare professionals to carry out bigger research in this field with more advanced inputs.

## Conclusion

To summarize, children with vascular ring KD-associated anomalies present early in life most of the time, but they suffer a delayed diagnosis due to the overlapping symptoms with common respiratory children illnesses. The use of imaging modalities; especially CTA is inevitable for definitive diagnosis and pre-operation planning. For the treatment, surgery has shown its effectiveness among other interventions, emphasizing adjunctive procedures as needed. Although post operative morbidity is not negligible and the risk of having persistent symptoms in some cases. In the end, there is a huge need of conducting more studies in larger, multi-centers with standardized diagnostic protocols and long term follow ups to refine surgical techniques needed and optimize strategies for full recovery.

## Data Availability

The raw data supporting the conclusions of this article will be made available by the authors, without undue reservation.
